# Digital anatomical study and clinical application of screw placement for quadrilateral plate fractures in the danger zone

**DOI:** 10.1186/s12891-020-03265-7

**Published:** 2020-04-11

**Authors:** Bei Zhao, Zhongye Sun, Wei Zhang, Zhongbao Xu, Xiaofei Yang, Weidong Mu

**Affiliations:** 1 Department of Traumatic Orthopaedics, Shandong Provincial Hospital Affiliated to Shandong University, 324 Jing Wu Road, Jinan, 250012 Shandong China; 2 Department of Orthopaedics, Liaocheng People’s Hospital, Liaocheng, Shandong China

**Keywords:** Acetabular fracture, Quadrilateral plate, Screw placement, Digital measurement

## Abstract

**Background:**

Direct screw placement for quadrilateral plate fractures in the danger zone of the acetabulum is very difficult. This study was performed to simulate the surgical procedure and try to obtain effective and safe screw angles through the middle window of the ilioinguinal approach in Chinese patients.

**Methods:**

We randomly collected the pelvic computed tomography (CT) scans of 100 adults. DICOM-formatted CT-scan images were imported into Mimics software. The three-dimensional reconstruction (3D) digital model of the semi-pelvis was established. A 3.5 mm cylinder was used to simulate the pathway of the screw from the designated insertion point. The angles of insertion and intersex differences were explored by statistical analyses.

**Results:**

The screws could be inserted via three angles: medial inclination, anterior inclination and posterior inclination. The mean minimum medial inclination angle (MIMIA) of insertion point A was 4.96° ± 1.11° in males and 8.66° ± 3.40° in females, and the intersex difference was significant. The mean minimum medial inclination angle (MIMIA) of insertion point B was − 5.31° ± 3.69° in males and 1.75° ± 8.95° in females, and the intersex difference was significant. There were no differences between any of the angles for males and females at insertion point O.

**Conclusions:**

Preoperative measurement and calculation by digital tools before screw placement for quadrilateral plate fractures of the acetabulum are feasible. Double cortical screws could be placed safely in the danger zone through the middle window of the ilioinguinal approach to increase the stability of the acetabulum.

## Background

The ilioinguinal approach is a standard anatomical approach that gives an excellent visual and exposure of the quadrilateral plate through the middle window [1]. Unfortunately, screw placement for acetabular fracture is difficult duo to its complicated geometry, especially when conducted in the danger zone of the acetabulum for the treatment of displaced quadrilateral plate fractures. Traumatic hip arthritis can be developed in patients with non-anatomically reduced fractures even with well-placed screws [2].

Many experts have studied the danger zone and have analysed screw implants in cadavers [3–5]. It is very difficult to place a double cortical screw directly in the danger zone to fix the quadrilateral plate [6, 7]. Several techniques have been reported to prevent complications with screw placement in the danger region of the acetabulum. However, these methods might increase operation time and cause iatrogenic trauma [8–10]. Therefore, finding accurate points of insertion, lengths and angles of screws are of high value before surgery [11].

To date, number of computer software related to orthopaedics have been reported to measure the safety of screw implantation [12, 13]. Materialise’s Interactive Medical Image Control System (Mimics), which is often used for three-dimensional reconstruction, simulating operations with the use of internal fixation and analysing data, might be a more efficient method for implants [14, 15]. Thus, we simulated the surgical procedure by using Mimics software and attempted to obtain safe and effective screw angles for the quadrilateral plate of the acetabulum in Chinese patients.

## Materials and methods

We retrospectively collected pelvic computed tomography (CT) scans (digital imaging and communication in medicine format) of 100 adults who had undergone continuous slice CT scanning at the imaging research centre of our hospital between September 2017 and February 2019. Patients were excluded if they had pelvic or acetabular fractures, tumours, severe deformities, or severe hip inflammation. This study was approved by the Institutional Review Board of our hospital, and patients’ informed consent was obtained. A total of 50 male and 50 female virtual pelvic models were created.

DICOM-formatted CT-scan images of each patient were imported into Mimics software (20.0; Materialise, Leuven, Belgium). We removed the soft tissue by image segmentation, removing the femoral head through region growth, and we removed the sacroiliac joint through multiple slice editing. The three-dimensional reconstruction (3D) digital model of the semi-pelvis was established.

**Fig. 1:**
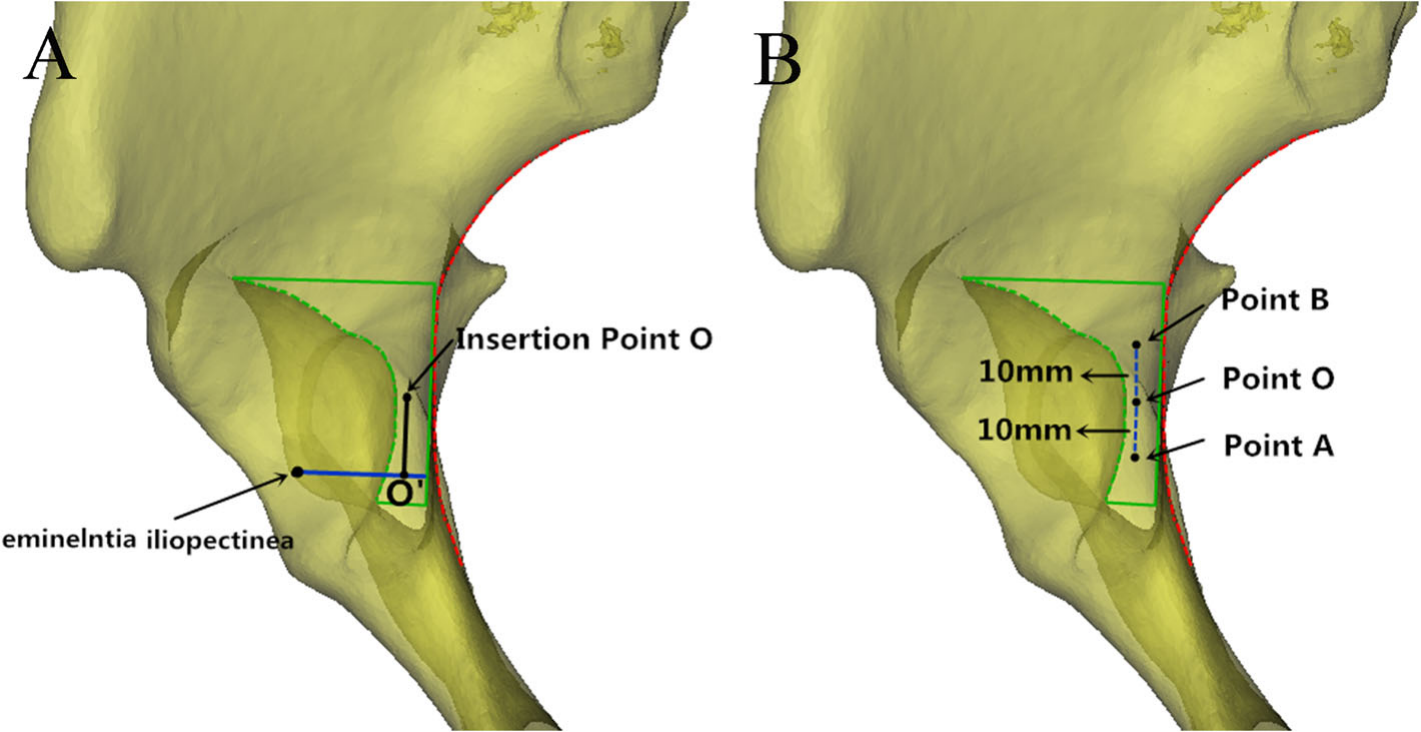
**a**: The axial observation perspective was parallel to the quadrilateral surface (green dotted line) and perpendicular to the arcuate line (red dotted line). The region surrounded by the green line was commonly used for screw placement through the middle window of the ilioinguinal approach. Insertion point O was 5 mm away from the quadrilateral surface. The blue line represents the distance from eminelntia iliopectinea to the quadrilateral surface. OO’ represents the distance from point O to the blue line. **b**: The distance (blue dotted line) from point A and point B to point O was 10 mm. Points A, O and B were on the same line, which was parallel to the quadrilateral surface

First, we reduced the transparency of the semi-pelvis models and observed the 3D model through the perspective method described in previous studies [16, 17]. According to the anatomical structure of the pelvis, the special area of the quadrilateral surface (under the arcuate line and behind the obturator foramen) is flat. The axial view should be parallel to this surface and perpendicular to the arcuate line. As shown in Fig. 1a, the region surrounded by the green line was commonly used for screw placement through the ilioinguinal approach. Under this visual angle, the inner border of the acetabulum was the green dotted line and the “most dangerous” screw insertion point was located on the shortest vertical line along the acetabular border to the quadrilateral surface. The width of the commonly used 3.5 mm curved reconstruction plate was 10 mm, so the insertion points for the screws were 5 mm away from the pelvic boundary. To facilitate operation and avoid deviation, the point O, 5 mm away from the quadrilateral surface, was considered the “most dangerous” screw insertion point. The distance from point A and point B to point O was 10 mm. Points A, O and B were on the same line, which was parallel to the quadrilateral surface (Fig. 1b). The key point of the study was to identify the location of screw insertion point O. As shown in Fig. 1a, the blue line initiated from the eminelntia iliopectinea was perpendicular to the quadrilateral surface. The distance from point O to the blue line was measured. It was marked as OO’.

**Fig. 2:**
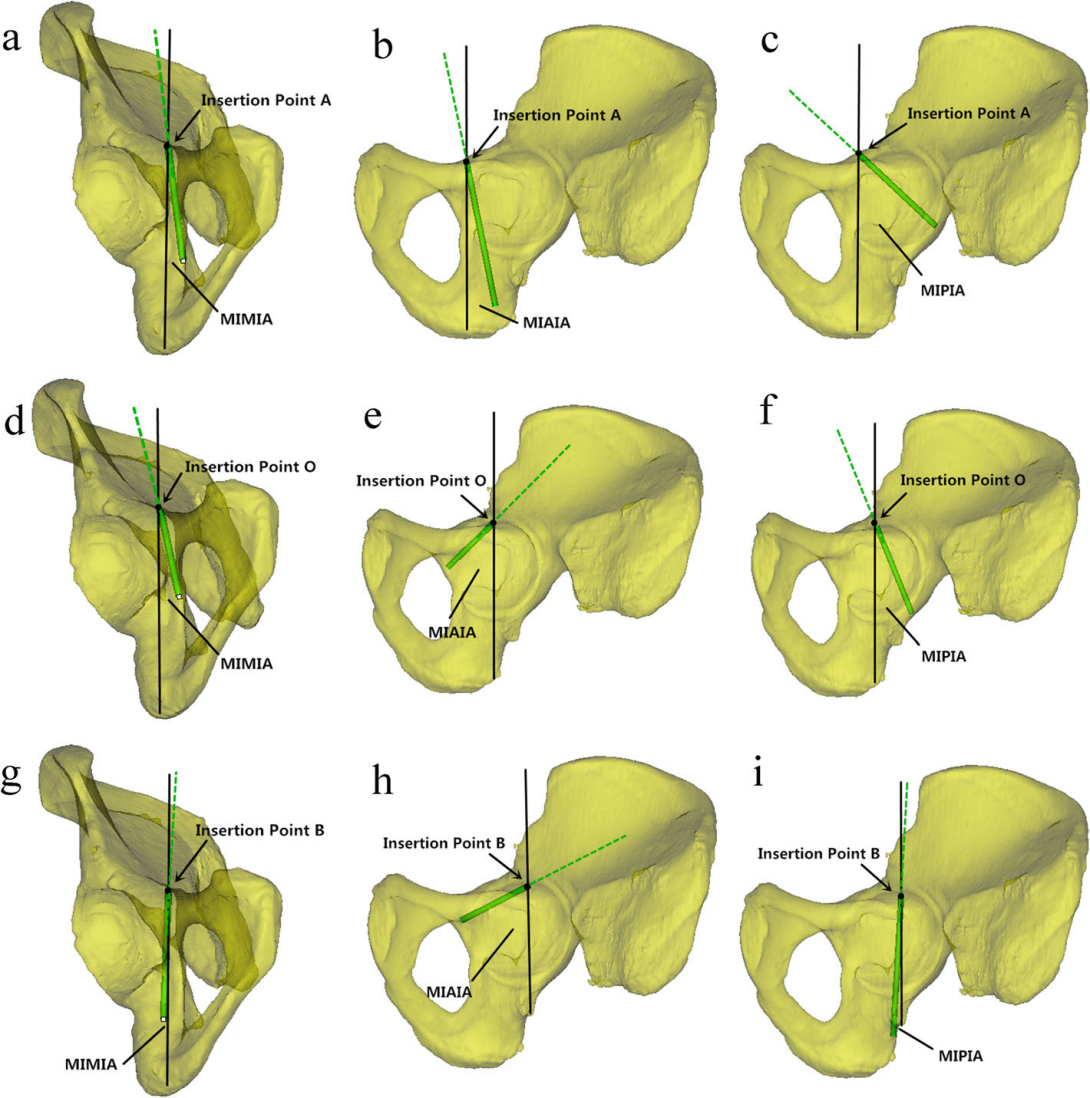
**a**-**i**: The illustration presenting the angles for safely introducing the drill-bit from Insertion Point A, O and B (simulating a clinical scenario): the angle between the green dotted line and the black solid line represents the angle of drill. With quadrilateral surface as a sign, we can see the range of three different angles in male and female

Then, 3.5 mm cylinders were employed to simulate the pathways of the screws from the insertion points A, O and B. The screws were in three directions: medial inclination (perpendicular to the arcuate line and pointing to the quadrilateral surface), anterior inclination and posterior inclination (parallel to the plane of the quadrilateral surface). In this procedure, screws were inserted tangential to the margins of the acetabular cortex, penetrating into the bilateral cortices (Fig. 2).

The angle between the reference line (black line: parallel to the quadrilateral surface and perpendicular to the arcuate line) and the screw tangential to the medial margin of the acetabular cortex was the minimum medial inclination angle (MIMIA). The angles between the reference line and the screws tangential to the anterior and posterior margins of the acetabular cortex were the minimum anterior inclination angle (MIAIA) and minimum posterior inclination angle (MIPIA), respectively. The MIMIA, MIAIA and MIPIA were measured (Fig. 2).

The collected data were analysed by SPSS 19.0 statistical software. The experimental data are represented as the mean ± SD. T tests were used to compare the data. Statistical significance was accepted at *p* < 0.05.

## Results

The study subjects included 50 males and 50 females aged between 18 and 79 years old, with a mean age of 53.03 ± 18.99 years. We observed that the screws could be inserted via three angles: the minimum medial inclination, anterior inclination and posterior inclination.

The distances between insertion point O and the reference blue line were 12.79 ± 3.41 mm in males, and 13.30 ± 3.21 mm in females. However, the value was not statistically significant for the different sexes (*P* > 0.05, Table 1).

**Table 1:** Measured distances (OO’) in different genders

Gender	L (mm)
Male(*n* = 50)	12.79 ± 3.41
Female(n = 50)	13.30 ± 3.21
t value*	−0.493
*P* value*	0.525

Note:* t and P are the results of inter-sex comparisons

As viewed from insertion point A, the mean MIMIA was 4.96° ± 1.11° in males and 8.66° ± 3.40° in females, and the intersex difference was significant (*P* < 0.05, Table 2). In males, the mean MIAIA and MIPIA were − 1.49° ± 6.29° and 45.75° ± 6.13°, respectively, from the view of insertion point A; in females, the values were − 1.81° ± 7.58° and 48.45° ± 14.69°, respectively (the negative value of the MIAIA indicated that the screw was placed in posterior inclination rather than in anterior inclination). However, the difference between the results for males and females was not statistically significant (*P* > 0.05, Table 2).

**Table 2:** Measured angles from insertion point A

Group	MIMIA(°)	MIAIA^#^(°)	MIPIA(°)
Male(*n* = 50)	4.96 ± 1.11	−1.49 ± 6.29	45.75 ± 6.13
Female(*n* = 50)	8.66 ± 3.40	1.81 ± 7.58	48.45 ± 14.69
t value*	− 3.260	−1.062	−0.535
*P* value*	0.014	0.344	0.602

Note:* t and P are the results of inter-sex comparisons. ^#^For the MIAIA, a negative value indicates a posterior inclination

As viewed from insertion point O, the insertion angle of the screw is recommended to be smaller in males than in females, but no significant differences were found in the inward tilt of the screws. In males, the mean MIAIA and MIPIA were 29.55° ± 10.21° and 25.74° ± 5.47°, respectively; in females, the values were 36.34° ± 11.16° and 30.45° ± 11.21°, respectively. These results did not significantly differ for the different sexes (P > 0.05, Table 3).

**Table 3:** Measured angles from insertion point O

Group	MIMIA(°)	MIAIA(°)	MIPIA(°)
Male(*n* = 50)	13.05 ± 2.67	29.55 ± 10.21	25.74 ± 5.47
Female(*n* = 50)	12.05 ± 2.24	36.34 ± 11.16	30.45 ± 11.21
t value*	0.904	−1.418	−1.193
*P* value*	0.537	0.576	0.058

Note:* t and P are the results of inter-sex comparisons

As viewed from insertion point B, the mean MIMIA was smaller in males than in females (− 5.31° ± 3.69° and 1.75° ± 8.95°, respectively, and the negative value of the MIMIA indicated that the screw was placed in lateral inclination rather than in medial inclination). The angle was significantly different for the different sexes (*P* < 0.05, Table 4). These results indicate that the screw at the line tangential to the acetabulum had more of an outward tilt angle in males than in females. The mean MIAIA and MIPIA were 56.14° ± 8.92° and − 5.12° ± 2.89°, respectively, in males; likewise, the angles were 61.21° ± 10.66° and 2.33° ± 5.20°, respectively, in females, (the negative value of the MIPIA indicated that the screw was placed in anterior inclination rather than in posterior inclination). The values for males and females were not significantly different (*P* > 0.05, Table 4).

**Table 4:** Measured angles from insertion point B

Group	MIMIA^#^ (°)	MIAIA(°)	MIPIA^#^ (°)
Male(*n* = 50)	−5.31 ± 3.69	56.14 ± 8.92	−5.12 ± 2.89
Female(*n* = 50)	1.75 ± 8.95	61.21 ± 10.66	2.33 ± 5.20
t value*	−2.304	−1.153	−3.952
*P* value*	0.008	0.856	0.101

Note:* t and P are the results of inter-sex comparisons. ^#^For the MIMIA, a negative value indicates a lateral inclination. For the MIPIA, a negative value indicates an anterior inclination

### Clinical cases

**Fig. 3:**
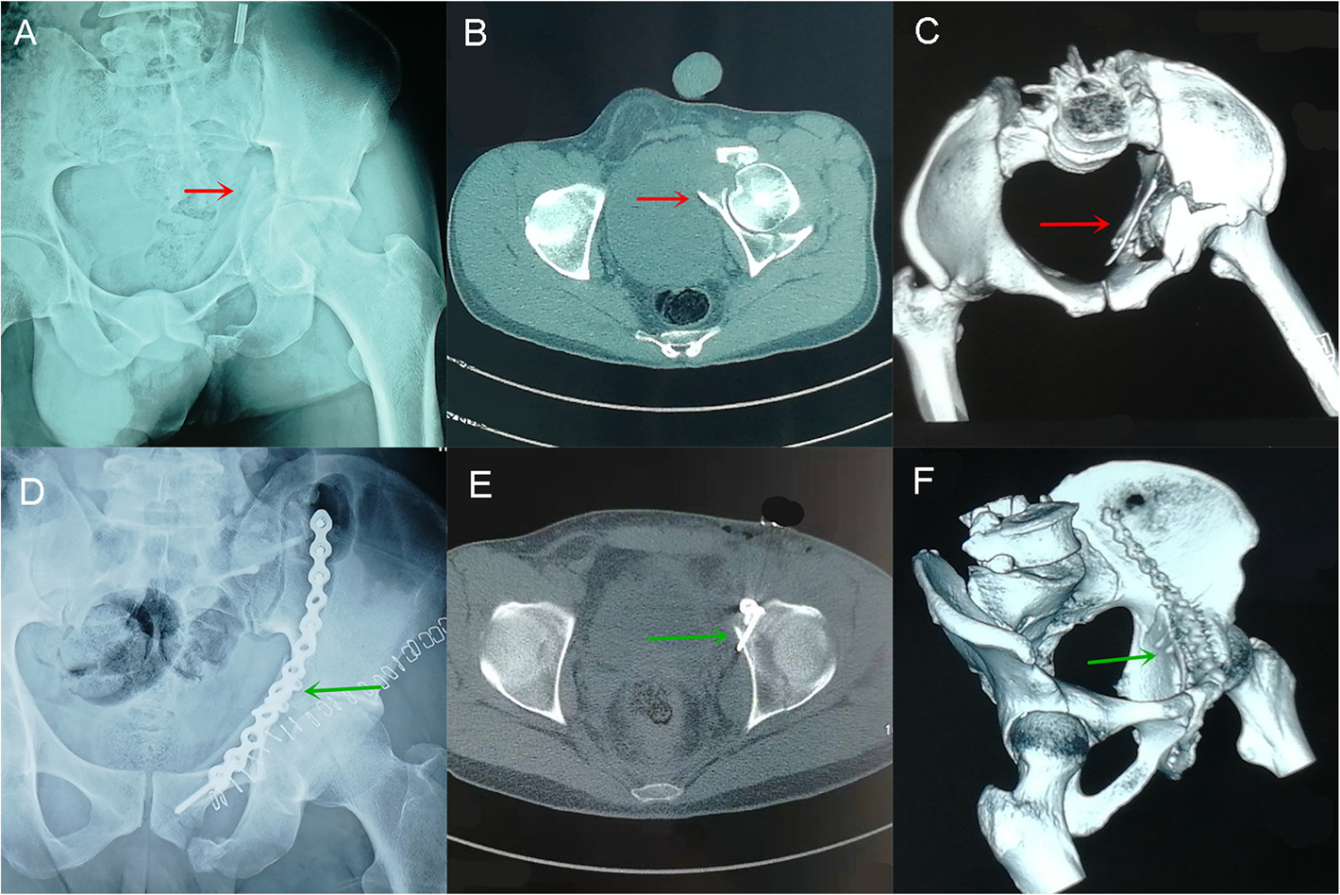
A 45-year-old man was injured by a car accident. **a-c**: preoperative X-Ray, CT cross section and 3D reconstruction. The red arrow represents the displaced quadrilateral plate fracture. **d-f**: postoperative X-Ray, CT cross section and 3D reconstruction. Green arrow shows two 3.5-mm cortical bone screws used to fix the quadrilateral plate

**Fig. 4:**
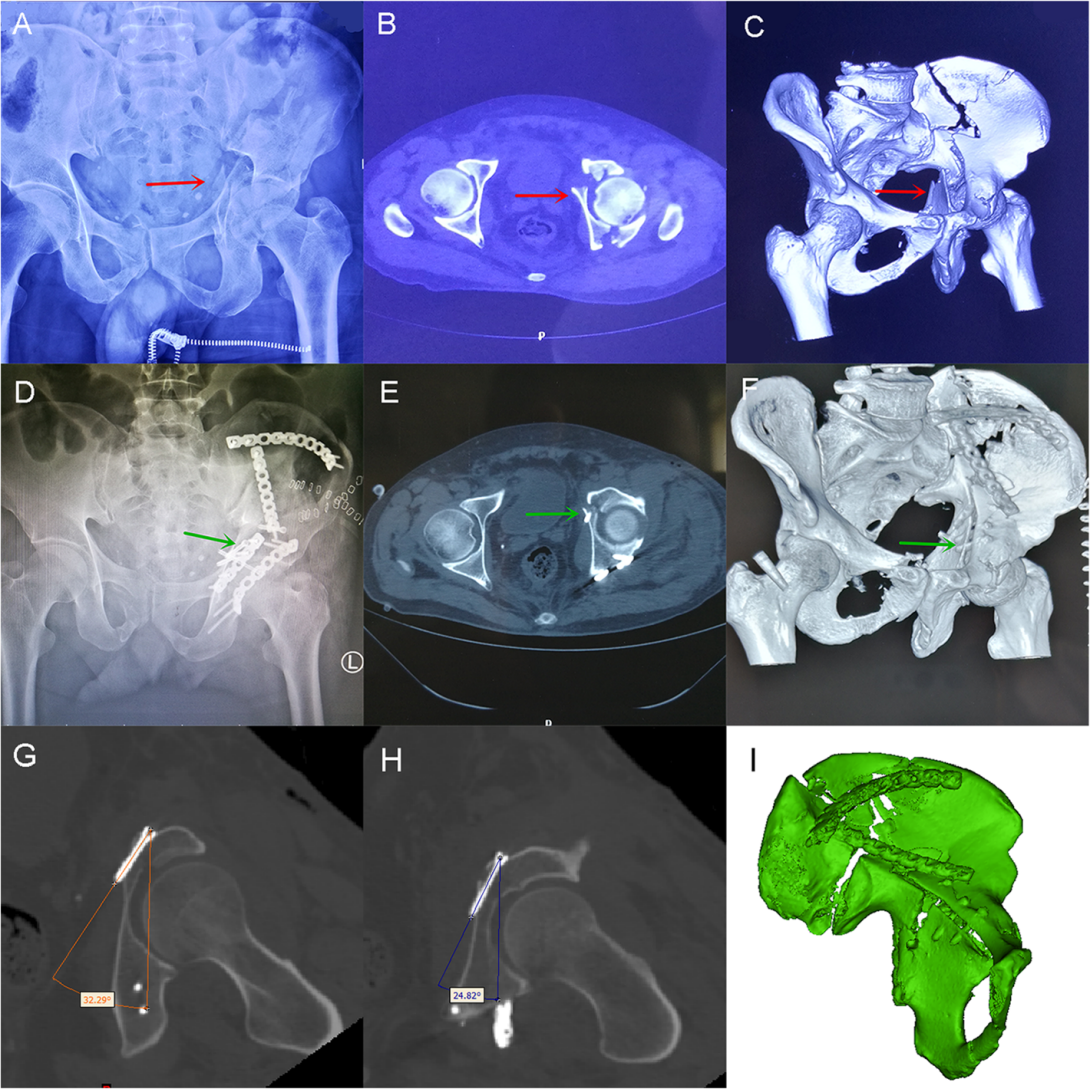
A 51-year-old man was suffered serious traffic injuries. **a-c**: preoperative X-Ray, CT cross section and 3D reconstruction. The red arrow represents the displaced quadrilateral plate fracture. **d-f**: postoperative X-Ray, CT cross section and 3D reconstruction. Green arrow shows two hollow countersunk screws used to fix the quadrilateral plate. **g-i**: Postoperative CT-scan images were imported into Mimics software. Observation of the coronal plane and 3D model of the semi-pelvis shows that the insertion angle of the screws corresponded to that of our study

As shown in Fig. 3, we can observe the three imaging pictures A-C from a 45-year-old man who was injured by a car accident. The red arrow represents the displaced quadrilateral plate fracture. After reduction, two 3.5-mm cortical bone screws were used to fasten the quadrilateral plate (green arrow shown in imaging D-F). Figure 4 shows X-ray and CT scans from a 51-year-old man who also suffered serious traffic injuries. We placed two hollow countersunk screws to fix the quadrilateral plate which also achieved a good reduction. Postoperative CT-scan images were imported into Mimics software. Observing from the coronal plane and 3D model of the semi-pelvis, we found that the insertion angle of the screws corresponded to that of our study (Fig. 4g-i).

## Discussion

Currently, the anterior ilioinguinal approach is commonly used in treating patients with pelvic and acetabular fractures [18, 19]. Internal fixation with a screw-plate system is currently the most frequent method of fixing acetabular fractures, and plate placement on the superior border of the arcuate line is commonly used [7, 20–22]. With the ilioinguinal approach, the area of the projection of the acetabulum onto the anterior surface can be exposed through the middle window [1].

The quadrilateral plate is an area similar to a trapezoid. It is bound by the obturator foramen anteriorly, the greater sciatic foramen posteriorly, the pelvic arcuate line superiorly, and the horizontal line joining the obturator foramen and the ischial spine inferiorly [23, 24]. Because of the special location of the quadrilateral plate, soft tissue structures are at risk when fixing quadrilateral plate fractures. Bleeding from the corona mortis, direct or indirect injury of obturator vessels and nerves, and direct injury to the urinary bladder by retractors are common complications [25, 26]. Quadrilateral plate fractures are mostly encountered in fractures of both columns, accompanied by central dislocations of the hip [7, 27]. In addition, T-type fractures, anterior column and posterior hemi-transverse fractures, posterior column and transverse fractures involve this inside wall [27, 28]. Open reduction and internal fixation with spring plates, H-shaped, T-shaped, L-shaped and reconstruction plates buttressing the quadrilateral surface, even with cerclage wires are common methods [29–31]. In some other studies, buttress plates have proven helpful in maintaining the quadrilateral surface or medial acetabular wall [21, 32]. However, the quadrilateral plate is too thin and is located in the danger zone, so these technologies include indirect or elastic fixation. In some studies, it is considered that the screws have to run parallel to the anterior border of the greater sciatic notch and parallel to the quadrilateral plate [1, 18, 33]. However, there are some limitations on the insertion point in clinical practice. Moreover, if the quadrilateral plate is too thin, we cannot place the screw in parallel and have to use a certain tilt angle. There is no systematic study in which screws are placed in the danger zone to directly fix the quadrilateral plate. In other words, it is very difficult to place a double cortical screw in the danger zone to fix the quadrilateral plate [7].

The combination of Mimics software and CT data not only saves manpower, materials and financial resources, but can also be repeated and verified by test results with high reliability in guiding practice [34]. At present, this combination is widely used in basic and clinical trial studies [35–37]. This digital anatomical measurement provides a reference for screw placement in pelvic and acetabular fractures. In this study, we confirmed the locations of three insertion points according to the location of the eminelntia iliopectinea and arcuate line. We studied the screw placement penetrating the double cortices via three different angles. As shown in Fig. 2, we could operate the drill-bit at safe angles from point A, O and B during the surgery.

In this study, we described that screw placement in the danger zone of the anterior surface of the acetabulum could be used as a common method for the treatment of acetabular fracture. Our results were analysed to find the ranges of effective and safe angles. Given the data obtained from our study, more screws could be applied to increase stability, without penetrating the acetabulum.

As shown in Tables 2 and 4, the MIMIA from insertion points A and B in males was significantly smaller than that in females. Consequently, it can be seen that females required a greater tilt towards the quadrilateral plate surface than males to avoid screw penetration of the joint. The reason for this may be that the thickness of the acetabular medial wall in this region was smaller in females than in males. There were no significant differences in angles between males and females in terms of point O, as shown in Table 3. We speculated that this could be due to the larger femoral head and wider acetabular margin in males, even though the thickness of the medial wall of the acetabulum was smaller in females.

Some related studies have reported screw placement in the danger zone of the acetabulum. Our data differ from those of some previous studies. Benedetti [38] selected the vertical line of the anterior surface of the anterior column as the reference for screw placement. However, this landmark is unreliable for the irregular outline. As the quadrilateral surface is relatively flat, it is very convenient to touch or mark its direction with a Kirschner wire. Therefore, in our study, we considered the quadrilateral surface as a reference to determine the angle of the screw, which could ensure the accuracy of the screw placement. In contrast to the research of Ji et al. [14], we performed a study of the anterior and posterior angles of screw placement. As shown in Fig. 2, the anterior and posterior inclination screws were long and able to penetrate into bilateral cortices. These screws are more stable than inward tilted screws and can be widely used in clinical practice. Confirmation of the screw insertion sites was decided by the holes on the steel plate in some related digital anatomical studies [34]. In our study, we determined the insertion points through the eminelntia iliopectinea. In the actual operation, 2 or more screws can be placed first to secure the reduction of quadrilateral plate fracture, as shown in Figs. 3 and 4. Then, the contoured plate can be inserted to strengthen the fixation.

Mimics software has been widely used in 3D reconstruction for the development of digital orthopaedics technology. Preoperative 3D modelling enables more effective diagnosis and simulates the surgical procedure [39–44]. Our study provides surgeons with accurate data for selecting the correct zones of entry and angles before the implantation of screws. Upper transverse and upper oblique fracture lines are often involved in acetabular fractures based on fracture mapping [45, 46], but we can place the screws into the quadrilateral plate and control the angle to ensure double cortices. The obstacle to applying the technique is the complex anatomical structure through the middle window of the ilioinguinal approach. The difficulty of exposure and the risk of neurovascular injury are problems we need to pay attention to and deal with. Perhaps we can further study the intraoperative guide tool to improve the technique.

There are some limitations to this study. Quadrilateral screws can be effectively applied to transverse and oblique fractures around the arcuate line. Nevertheless, they play a limited role in severe comminuted fractures or lower fracture lines. We only studied the fixation of the fracture. However, how to perform fracture reduction was not mentioned. We only researched the common method of screw placement (superior border of the arcuate line). In addition, more patterns of fixation should be considered. We only studied the pelvises of Chinese people, who have different skeletal shapes than the American and European populations. In addition, we did not collect data according to height, weight or body bone density. Moreover, during the actual operation, we had to account for not only the bone but also the soft tissue. These factors may affect the implantation of screws.

## Conclusion

Our results imply that the direct implantation of screws for quadrilateral plate fractures in the danger zone of the acetabulum with the use of preoperative measurements and calculations by digital tools is feasible. Mimics software can be used for 3D reconstruction, imitating screw implantation, and reducing unnecessary injury during operation. Double cortical screws could be placed stably and safely through the middle window of the ilioinguinal approach.

## Data Availability

All relevant data was presented within the manuscript and the datasets used and/or analyzed during the current study are available from the corresponding author on reasonable request.

## Abbreviations

CT: Computed tomography
3D: Three-dimensional reconstruction
MIMIA: Minimum medial inclination angle
MIAIA: Minimum anterior inclination angle
MIPIA: Minimum posterior inclination angle
Mimics: Materialise’s Interactive Medical Image Control System

## References

[1] Fensky F, Lehmann W, Ruecker A (2018). Ilioinguinal approach [J]. J Orthop Trauma.

[2] Kacra BK, Arazi M, Cicekcibasi AE (2011). Modified medial Stoppa approach for acetabular fractures: an anatomic study.[J]. J Trauma Acute Care Surg.

[3] Ebraheim NA, Waldrop J, Yeasting RA (1992). Danger zone of the acetabulum.[J]. J Orthop Trauma.

[4] Xu R, Ebraheim NA, Farooq A (1997). Placement of screws in the uncemented acetabulum: anatomic analysis of the danger zone.[J]. Orthopedics.

[5] Wang XQ, Zhang W, Sun S (2006). Anatomic study of internal fixation of acetabular anterior column plate technique.[J]. Zhonghua wai ke za zhi.

[6] Guy P, Ej AOM, Helmy N (2010). The 'safe zone' for extra-articular screw placement during intra-pelvic acetabular surgery.[J]. J Orthop Trauma.

[7] White G, Kanakaris NK, Faour O (2013). Quadrilateral plate fractures of the acetabulum: an update [J]. Injury.

[8] Anglen JO, DiPasquale T (1994). The reliability of detecting screw penetration of the acetabulum by intraoperative auscultation. J Orthop Trauma.

[9] Ebraheim NA, Savolaine ER, Hoeflinger MJ, Jackson WT (1989). Radiological diagnosis of screw penetration of the hip joint in acetabular fracture reconstruction. J Orthop Trauma.

[10] Carmack DB, Moed BR, McCarroll K, Freccero D (2001). Accuracy of detecting screw penetration of the acetabulum with intraoperative fluoroscopy and computed tomography. J Bone Joint Surg Am.

[11] Caviglia H, Mejail A, Landro ME (2018). Percutaneous fixation of acetabular fractures. EFORT Open Rev.

[12] Zhang S, Su W, Luo Q (2014). Measurement of the “safe zone” and the “dangerous zone” for the screw placement on the quadrilateral surface in the treatment of pelvic and acetabular fractures with stoppa approach by computational 3D technology. Biomed Res Int.

[13] Ji X, Bi C, Wang F (2015). Digital anatomical measurements of safe screw placement at superior border of the arcuate line for acetabular fractures. BMC Musculoskelet Disord.

[14] Jiantao L, Hao Z, Peng Y (2015). A new measurement technique of the characteristics of nutrient artery canals in tibias using Materialise’s interactive medical image control system software [J]. Biomed Res Int.

[15] Tang M, Yin Z, Morris SF (2008). A pilot study on three-dimensional visualization of perforator flaps by using angiography in cadavers [J]. Plast Reconstr Surg.

[16] Feng X, Zhang S, Luo Q (2016). Definition of a safe zone for antegrade lag screw fixation of fracture of posterior column of the acetabulum by 3D technology [J]. Injury.

[17] Feng X, Fang J, Lin C (2015). Axial perspective to find the largest intraosseous space available for percutaneous screw fixation of fractures of the acetabular anterior column [J]. Int J Comput Assist Radiol Surg.

[18] Matta JM (1994). Operative treatment of acetabular fractures through the ilioinguinal approach: a 10-year perspective. Clin Orthop Relat Res.

[19] Stöckle U, Hoffmann R, Südkamp NP, Reindl R, Haas NP (2002). Treatment of complex acetabular fractures through a modifed extended iliofemoral approach. J Orthop Trauma.

[20] Uchida K, Kokubo Y (2013). Takafumi Yayama, et al. fracture of the acetabulum: a retrospective review of ninety-one patients treated at a single institution [J]. Eur J Orthopaedic Surg Traumatology.

[21] Karim MA, Abdelazeem AH, Youness M (2017). Fixation of quadrilateral plate fractures of the acetabulum using the buttress screw: a novel technique [J]. Injury.

[22] Hirvensalo E, Lindahl J, Kiljunen V (2007). Modified and new approaches for pelvic and acetabular surgery. Injury..

[23] Elnahal WA, Karim MA, Khaled SA, et al. Quadrilateral Plate Fractures of the Acetabulum: Proposition for a Novel Classification System [J]. Injury. 2017:49(2). 10.1016/j.injury.2017.11.041 29241997

[24] Laflamme GY, Delisle J, Leduc S (2009). Isolated quadrilateral plate fracture: an unusual acetabular fracture [J]. Can J Surg.

[25] Khoury A, Weill Y, Mosheiff R (2012). The Stoppa approach for acetabular fracture [J]. Oper Orthop Traumatol.

[26] Schwabe P, Wichlas F, Druschel C (2014). Complications after osteosynthetic treatment of acetabular fractures [J]. Orthopäde.

[27] Laflamme GY, Hebert-Davies J, Rouleau D (2011). Internal fixation of osteopenic acetabular fractures involving the quadrilateral plate [J]. Injury-Int J Care Injured.

[28] Qureshi AA, Archdeacon MT, Jenkins MA (2004). Infrapectineal plating for Acetabular fractures: a technical adjunct to internal fixation [J]. J Orthop Trauma.

[29] Matta JM, Mehne DK, Roffi R (1986). Fractures of the acetabulum. Early results of a prospective study.[J]. Clin Orthop Relat Res.

[30] Giannoudis PV, Grotz MRW, Papakostidis C (2005). Operative treatment of displaced fractures of the acetabulum [J]. China J Orthopaedics Traumatology.

[31] Makwana K, Vijayvargiya M, Agarwal N (2018). A rare case of bilateral central subluxation of the hip joint with associated bilateral quadrilateral plate fracture in an elderly male due to seizure activity [J]. Rev Bra Orto.

[32] Peter RE (2015). Open reduction and internal fixation of osteoporotic acetabular fractures through the Ilio-inguinal approach: use of buttress plates to control medial displacement of the quadrilateral surface [J]. Injury.

[33] Kaifang C, Yanhui J, Zhenfei H, et al. A single modified ilioinguinal approach for the treatment of acetabular fractures involving both columns [J]. J Orthop Trauma. 2018;1. 10.1097/BOT.0000000000001303 30138151

[34] Li W, Zhao F, Sun Z (2018). Digital anatomy to improve screw insertion techniques for plate-screw fixation of the pubic body. Biomed Res Int.

[35] Popat H, Richmond S, Benedikt L (2009). Quantitative analysis of facial movement-a review of three-dimension imaging techniques. Comput Med Imaging Graph.

[36] Guo H, Wang Y, Dai J (2018). Application of 3D printing in the surgical planning of hypertrophic obstructive cardiomyopathy and physician-patient communication: a preliminaiy study. J Thorac Dis.

[37] Chang S, Yang Y, Liu Y (2018). How Does the Remodeling Capacity of Children Affect the Morphologic Changes of Fractured Mandibular Condylar Processes After Conservative Treatment?. J Oral Maxillofac Surg.

[38] Benedetti JA, Ebraheim NA, Xu R (1996). Anatomic considerations of plate-screw fixation of the anterior column of the acetabulum. J Orthop Trauma.

[39] Chana Rodríguez F, Pérez Mananes R, Narbona Cárceles FJ (2018). 3D printing utility for surgical treatment of acetabular fractures [J]. Rev Esp Cir Ortop Traumatol.

[40] Yu AW, Duncan JM, Daurka JS (2015). A feasibility study into the use of three-dimensional printer Modelling in Acetabular fracture surgery [J]. Advances Orthopedics.

[41] Duncan JM, Samuel N, Kashif A (2015). The use of a 3D printer in pre-operative planning for a patient requiring Acetabular reconstructive surgery:[J]. J Orthopaedic Case Rep.

[42] Mitchell S (2014). Tactile surgical navigation system for complex acetabular fracture surgery.[J]. Orthopedics.

[43] Chen X, Chen X, Zhang G (2017). Accurate fixation of plates and screws for the treatment of acetabular fractures using 3D-printed guiding templates: an experimental study [J]. Injury.

[44] Lalit M, Abhishek M, Gaurang A (2018). 3D printing in designing of anatomical posterior column plate [J]. J Clin Orthopaedics Trauma.

[45] Yun Y, Min Y, Chang Z (2018). Mapping of 238 quadrilateral plate fractures with three-dimensional computed tomography [J]. Injury.

[46] Yun Y, Chang Z, Yue F (2019). A study on fracture lines of the quadrilateral plate based on fracture mapping [J]. J Orthop Surg Res.

